# Cognition-Mortality Associations Are Stronger When Estimated Jointly in Longitudinal and Time-to-Event Models

**DOI:** 10.1093/geroni/igab046.1196

**Published:** 2021-12-17

**Authors:** Stephen Aichele, Sezen Cekic, Patrick Rabbitt, Paolo Ghisletta

**Affiliations:** 1 Colorado State University, Fort Collins, Colorado, United States; 2 University of Geneva, University of Geneva, Geneve, Switzerland; 3 University of Oxford, University of Oxford, England, United Kingdom

## Abstract

Objectives: With aging populations worldwide, there is growing interest in links between cognitive decline and elevated mortality risk—and, by extension, analytic approaches to further clarify these associations. Toward this end, some researchers have compared cognitive trajectories of survivors vs. decedents while others have examined longitudinal changes in cognition as predictive of mortality risk. A two-stage modeling framework is typically used in this latter approach; however, several recent studies have used joint longitudinal-survival modeling (i.e., estimating longitudinal change in cognition conditionally on mortality risk, and vice versa). Methodological differences inherent to these approaches may influence estimates of cognitive decline and cognition-mortality associations. These effects may vary across cognitive domains insofar as changes in broad fluid and crystallized abilities are differentially sensitive to aging and mortality risk. Methods: We applied each of the above analytic approaches to data from a large-sample repeated-measures study of older adults (N = 5,954, of whom 4,453 deceased; ages 50–87 years at assessment). Results: Cognitive trajectories indicated worse performance in decedents and when estimated jointly with mortality risk, but this was attenuated after adjustment for health-related covariates. Better cognitive performance predicted lower mortality risk, and, importantly, cognition-mortality associations were stronger when estimated in joint models. Associations between mortality risk and crystallized abilities only emerged under joint estimation, confirming the greater power of this statistical approach. Discussion: These results suggest that joint estimation of cognition-mortality associations may be beneficial for research in cognitive epidemiology and cognitive reserve in adult development.

